# Secondary metabolites from the *Aspergillus* sp. in the rhizosphere soil of *Phoenix dactylifera* (Palm tree)

**DOI:** 10.1186/s13065-019-0624-5

**Published:** 2019-08-07

**Authors:** Raha Orfali, Shagufta Perveen

**Affiliations:** Department of Pharmacognosy, College of Pharmacy, King Saud University, PO Box 2457, Riyadh, 11451 Saudi Arabia

**Keywords:** *Aspergillus* sp., Rhizosphere fungi, Antimicrobial activity, *Phoenix dactylifera*

## Abstract

The soil-derived fungus *Aspergillus* sp. isolated from the rhizospheric soil of *Phoenix dactylifera* (Date palm tree) and cultured on the large scale solid rice medium yielded a novel compound 1-(4-hydroxy-2,6-dimethoxy-3,5-dimethylphenyl)-2-methyl-1-butanone (**1**) and four known compounds; citricin (**2**), dihydrocitrinone (**3**), 2, 3, 4-trimethyl-5, 7-dihydroxy-2, 3-dihydrobenzofuran (**4**) and oricinol (**5**). The structures of the isolated compounds were elucidated by MS, 1H, 13C and 2D NMR spectra. Compound (**1**) exhibited potent antimicrobial activities against *Staphylococcus aureus* with MIC values of 2.3 μg mL^−1^ and significant growth inhibitions of 82.3 ± 3.3 against *Candida albicans* and of 79.2 ± 2.6 against *Candida parapsilosis*. This is the first report to isolate metabolites from the fungus *Aspergillus* found in temperate region date plant rhizospheres.

**Electronic supplementary material:**

The online version of this article (10.1186/s13065-019-0624-5) contains supplementary material, which is available to authorized users.

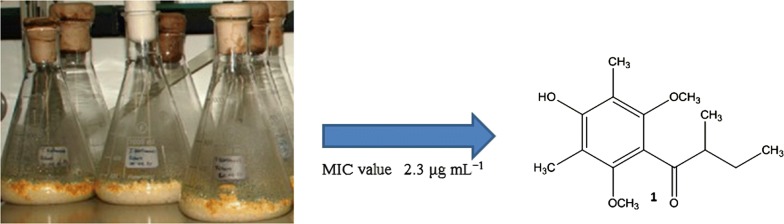

## Introduction

The rhizosphere is the portion of the soil which is surrounding the plant root [1, 2]. This soil inhabited a great microbial diversity than nonrhizosphere soil [3].The microorganisms in the rhizosphere play a great biological role in the growth of host plant. This occurs through the defense mechanism provided by the rhizosphere microbial communities against pathogens or through providing nutrition to the plant by their role in mineralization of different organic compounds [4, 5]. Fungi for instance, provide the plant with phosphorous while asymbiotic and symbiotic bacteria play an important role in nitrogen fixation and instantly increase of the available nitrogen in the rhizosphere region [6]. However, the diversity of microbial strains varies from one rhizosphere to another according the species of the plant and the environmental factors [7, 8].

Recent reports show that the rhizosphere region of soil hills is untapped source of clinically important microorganisms, especially fungi [9–14] which produce a large number of bioactive metabolites. However, the attention for isolation of novel compounds with great pharmaceutical value from this fungal habitat still limited comparing to endophytes and marine niches.

*Phoenix dactylifera*, usually known as a date palm tree, it is globally valued for its health and nutritional-promoting fruit [15]. This tree grown in the arid and semi-arid regions especially areas which have long, dry summer and mild winter are best for date palm cultivation [16]. Kingdom of Saudi Arabia is the second top producer and exporter of dates since this tree covers more than 170 thousand hectares [17].

The filamentous fungi *Aspergillus* are ubiquitous opportunistic moulds that are pathologically and therapeutically important [18]. Many literatures reported numerous bioactive metabolites isolated from *Aspergillus* sp. [19–21]. These metabolites showed significance therapeutic importance such as anticancer and antimicrobial activities. The biological value of this fungal species, make it of considerable interest to the scientific research community for discovering further novel bioactive compounds [22].

As a part of our ongoing search on bioactive fungal secondary metabolites from unexplored niches [23, 24], in this study, a fungal strain RO-17-3-2-4-1, identified as *Aspergillus* sp., was isolated from the rhizosphere soil of *P. dactylifera*, Wadi Hanifa, 15 km Northwest of Riyadh, Saudi Arabia. To the best of our knowledge, it is the first research report on the isolation of secondary metabolites from the rizosphere soil of temperate region plants *P. dactylifera*.

## Results and discussion

### Isolation and structural identification

**Fig. 1:**
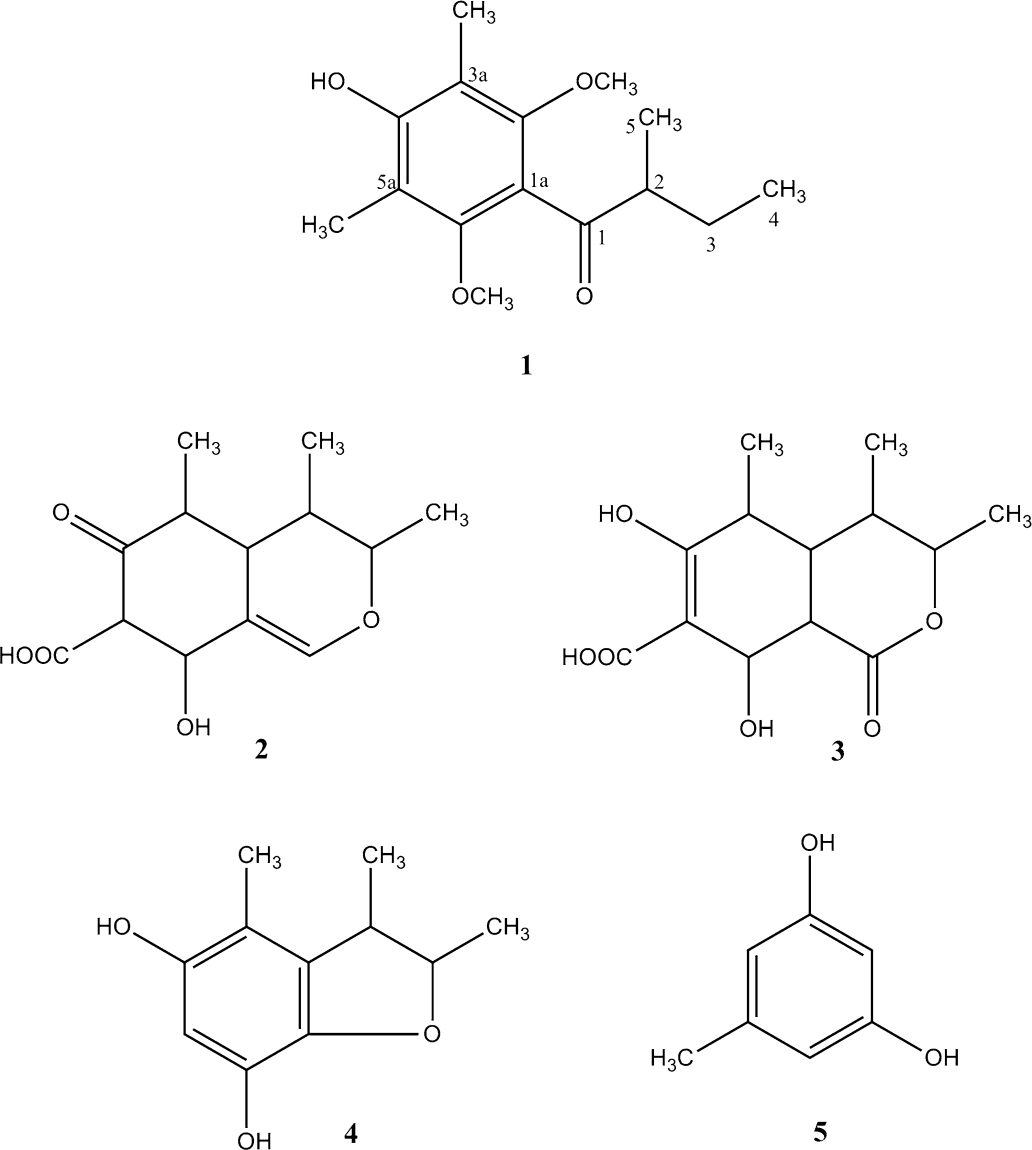
Structures of compounds **1**–**5**

Disease suppressive soils offer effective protection to plants against infection by soil borne pathogens. Therefore, suppressive soils are considered as a rich source for the discovery of microorganisms which provides novel secondary metabolites on large scale culture. To date, a plethora of work has been done on the fungal culture of the obtained microorganism from these soils which led to the isolation of novel biologically active constituents. In our ongoing research on the findings of soil based microorganism and its culture for the identification of secondary metabolites, we worked on the crude ethyl acetate extract of the interrhizospheric fungus (*Aspergillus* sp.). It exhibited considerable antimicrobial activity against the tested bacterial and fungal strains. Bioactivity-guided fractionation led to the isolation of one new compound 1-(4-hydroxy-2,6-dimethoxy-3,5-dimethylphenyl)-2-methyl-1-butanone **1**, together with four known compounds; citricin **2**, dihydrocitrinone **3**, 2, 3, 4-trimethyl-5, 7-dihydroxy-2, 3-dihydrobenzofuran **4**, and oricinol **5** (Fig. 1). Herein, we report the structure elucidation and biological evaluation of the isolated compounds.

**Fig. 2:**
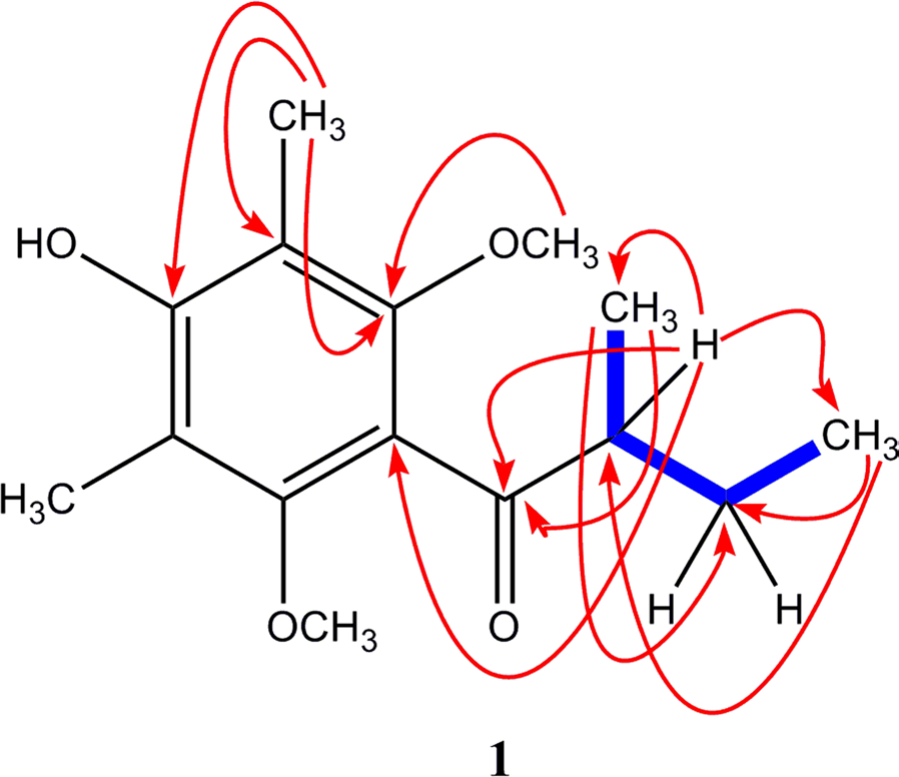
The key HMBC ( → ) & ^1^-^1^H COSY (blue solid line) correlations of compound **1**

The molecular formula of compound **1** was established to be C_15_H_22_O_4_ by ^1^H and ^13^C NMR spectroscopic data and ( ± ) HRESIMS. The ^1^H NMR data of **1** exhibited signals for four methyl protons at δH 0.85 (t, 7.7 Hz, CH_3_-4), 1.02 (d, 7.0 Hz, CH_3_-5), and 2.04 (s, CH_3_-3a and 5a); two methoxy groups at δH 3.57 (s, 2a and 6a-OCH_3_); one methylene protons at δH 1.27 (ddd, 7.0, 7.7, 14.0 Hz, H-3) and 1.63 (ddd, 7.0, 7.7, 14.0 Hz, H-3) and one methine proton at δH 2.81 (ddd, 7.0, 14.0 Hz, H-2). The ^13^C NMR data of **1** showed fifteen carbon signals, corresponding to one carbonyl carbon, six aromatic carbons (non-protonated carbons), two methoxy carbons, one methylene carbon, one methine carbon and four methyl carbons. These NMR signals suggested that compound **1** has fully substituted aromatic ring with butanone side chain, which was confirmed by long range HMBC correlations (Fig. 2). The methine proton at δH 2.81 (H-2) showed ^3^*J* HMBC correlation with the aromatic carbon at δC 122.2 (C-1a) and methyl carbon at 11.8 (C-4), while ^2^*J* correlation with carbonyl carbon at δC 207.9 (C-1), methylene carbon δC 25.3 (C-3) and methyl carbon 15.6 (C-5). The methoxy protons at δH 3.57 showed ^3^* J* HMBC correlations with the carbon at δC 153.7 (C-2a & 6a), indicated that methoxy groups were attached to the C-2a and C-6a of the aromatic ring, respectively. The two methyl groups appeared relatively low field in ^1^H NMR at δH 2.04 (6H, s), while high field in carbon ^13^C NMR δC 9.7 which confirmed its attachment at aromatic ring. This attachment was further confirmed by ^2^*J* HMBC correlations of methyl protons at δH 2.04 to the quaternary carbon at δC 114.3 (C-3a & 5a). The low field carbon resonance at δC 156.0 confirmed the presence of one hydroxyl group at aromatic ring which was assumed to be attached to C-4a. The adjacent position of hydroxyl and methyl group at aromatic ring was further confirmed through the ^3^*J* HMBC correlations of methyl protons (δH 2.04) to the hydroxyl bearing quaternary carbon at δC 156.0 (C-4a). Thus, the structure of compound **1** was assigned as 1-(4-hydroxy-2,6-dimethoxy-3,5-dimethylphenyl)-2-methyl-1-butanone.

The known compounds were identified as citricin **2** [25], dihydrocitrinone **3** [25], 2, 3, 4-trimethyl-5, 7-dihydroxy-2, 3-dihydrobenzofuran **4** [26], and oricinol **5** [27], through comparison.

of the NMR data with literature values.

### Biological activities

All isolated compounds (**1**–**5**) were evaluated for their antimicrobial activity against pathogenic bacteria and fungi by disc diffusion method by measuring the inhibition zones and for the active compounds (MIC) minimum inhibitory concentration values were also determined. Interesting antimicrobial properties were observed (Table 1), showed that compound **1** had antibacterial activities against *Staphylococcus aureus* with MIC values of 2.3 μg mL^−1^. Followed by compound **4** which recorded MIC of 15.6 μg mL^−1^against *Staphylococcus aureus.* Compound **1** further showed strong activity against the pathogenic bacteria *Escherichia fergusonii* with MIC of 3.1 μg mL^−1^. For human pathogenic fungi, the simple aromatic compound **5** disclosed the most significant growth inhibitions of 92 ± 3.9 and 90 ± 2.8 at 50 μg mL^−1^ against *Candida albicans* and *Candida parapsilosis,* respectively. Followed by compounds **1**, **2**, and **4** with higher inhibition value than the positive control Itraconazole a broad-spectrum antifungal drug. Compounds **3** neither showed antifungal nor antibacterial activity at 25 μg mL^−1^. These result suggested that the aromatic ring in polyketides may strengthen the antibacterial and antifungal activities of this class of compounds.

**Table 1:** In vitro antimicrobial activities of compounds **1–5**

Compound	Growth inhibition (%, mean ± SD)^a^				MIC (lg mL^−1^)^b^		
	*Candida albicans*	*Candida parapsilosis*	*S. aureus*	*B. licheniformis*	*E. xiangfangensis*	*E. fergusonii*	*P. aeruginosa*
**1**	82.3 ± 3.3	79.2 ± 2.6	2.3	> 25	> 25	3.1	> 25
**2**	67.3 ± 2.1	72.2 ± 2.8	> 25	> 25	> 25	> 25	> 25
**3**	23.6 ± 5.2	18.9 ± 3.7	> 25	> 25	> 25	> 25	> 25
**4**	61.2 ± 3.3	69.5 ± 2.4	15.6	> 25	> 25	> 25	> 25
**5**	92 ± 3.9	90 ± 2.8	> 25	> 25	> 25	> 25	> 25
Itraconazole	54.7 ± 2.6	51.5 ± 4.1	**_**	**_**	_	_	_
Amikacin	_	**_**	0.523	0.523	0.523	0.523	0.523

^a^Results expressed as mean ± standard deviation (SD)

^b^MIC > 25 μg mL^−1^

## Experimental

### General experimental procedures

The experimental procedure has written in Additional file 1.

### Plant and fungal strain materials

The fungal strain was isolated from rhizosphere soil of *P. dactylifera*, Wadi Hanifa, 15 km Northwest of Riyadh, KSA, in October 2017 and deposited in the laboratory of Pharmacognosy department, KSU. The fungus was identified as *Aspergulis* sp. (GenBank accession No. MK028999) according to DNA amplification sequencing of the fungal ITS region as reported in literature [28, 29].

### Fermentation, extraction and isolation

**Fig. 3:**
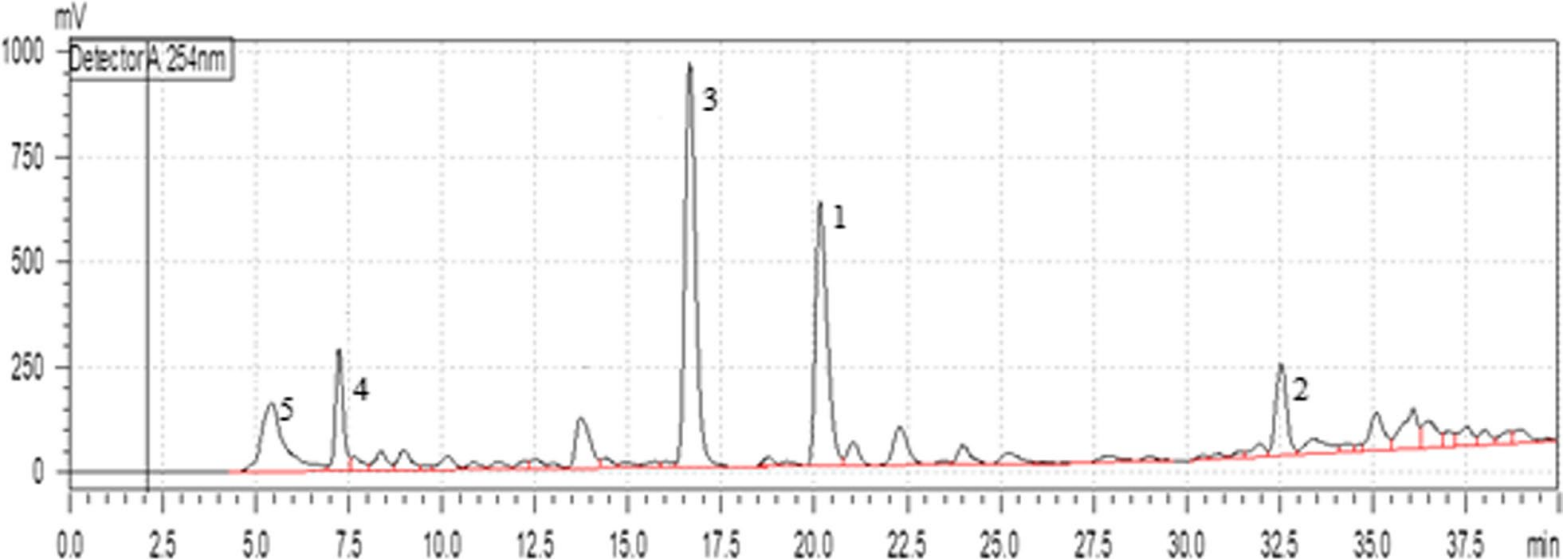
The HPLC chromatogram for ASLVLCS-4

The fungal strain was cultivated on both Wickerham liquid medium ASL (Yeast 3.0 g, Malt 3.0 g, Peptone 5.0 g, and Glucose 10.0 g in 1000 ml distilled water) and solid rice medium ASS prepared by autoclaving 100 g of commercially available milk rice and 100 mL of water in a 1 L Erlenmeyer flask. The flasks were autoclaved at 121 °C for 20 min and then cooled to room temperature. The strain RO-17-3-2-4-1 was grown in a constant temperature incubator at 20 °C under static conditions with shaking (180 rpm). The crude ethyl acetate extract of ASL (80 mg) harvested at 14 d and ASS (100 mg) harvested at 20 d were subjected to antimicrobial and HPLC analysis. After evaluation of the aforementioned data, the fungal strain further cultivated on solid rice medium and fermented in fifteen 1L Erlenmeyer flasks. After 21 days, full fungal growth was noticed and each flask was extracted overnight with ethyl acetate (3 × 500 mL), followed by filtration and evaporation. The obtained crude extract (8.0 g) was then partitioned between *n*-hexane and 90% aqueous MeOH. The MeOH extract was then subjected to vacuum liquid chromatography (VLC) on silica gel 60 using a gradient elution solvent system of *n*-hexane–EtOAc (100:0 to 0:100) and CH_2_Cl_2_–MeOH (100:0 to 0:100), where an eluting volume of 1000 mL was collected for each step, yielding twelve sub-fractions (ASVLC1-12). Sub-fraction (ASVLC.2) (1.0 g) was chromatographed on a Sephadex LH-20 column (100 × 2.5 cm) using 100% methanol as an eluting solvent. After combining similar fractions, six subtractions were obtained and fraction (ASVLCS 4) (Fig. 3) were chosen for further purification using semi-preparative HPLC with a gradient of MeOH/H_2_O as eluent system to afford **1** (3.2 mg), **2** (3.3 mg) **3** (5.1 mg), **4** (3.6 mg) and **5** (2.0 mg).

#### 1-(4-Hydroxy-2,6-dimethoxy-3,5-dimethylphenyl)-2′-methyl-1′-butanone (1)

Yellow gummy solid; [α]^25^_D_ + 34 (c = 0.05, MeOH); ^1^H-NMR (700 MHz, DMSO) and ^13^C-NMR (175 MHz, DMSO) spectroscopy data: see Table 2. ESIMS: Negative-ion mode *m/z* 265.1514 [M−H]^−^ (calcd for C_15_H_21_O_4_, 265.1439); Positive-ion mode *m/z* 267.11677 [M + H]^+^ (calcd for C_15_H_23_O_4_, 267.1596).

**Table 2:** ^1^H and ^13^C NMR spectroscopic data of compound **1**

No. #	**1**	
	δH	δC
1a	–	122.2
2a & 6a	–	153.7
3a & 5a	–	114.3
	–	
4a	–	156.0
5a	–	
6a	–	
1	–	207.9
2	2.81 ddd (7.0, 14.0)	48.6
3	1.27 ddd (7.0, 7.7, 14.0)	25.3
	1.63 ddd (7.0, 7.7, 14.0)	
4	0.85 t (7.7)	11.8
3a & 5aCH_3_	2.04 s	9.7
2a & 6a OCH_3_	3.57 s	62.5
5-CH_3_	1.02 d (7.0)	15.6

(^1^H NMR 700 MHz ^13^C NMR 175 MHz, δ in ppm, *J* coupling constants is in Hz)

### Antibacterial assay

The antibacterial activity was determined according the reported method [20]. The Gram-positive, *Staphylococcus aureus* (CP011526.1) and *Bacillus licheniformis* (KX785171.1) and the Gram-negative, *Enterobacter xiangfangensis* (CP017183.1)*, Escherichia fergusonii* (CU928158.2) and *Pseudomonas aeruginosa* (NR-117678.1) bacteria were suspended in a nutrient broth for 24 h then spread on Muller Hinton agar plate. 10 µL of the sample solution were loaded in wells using Amikacin as positive control. The clear area which was free of microbial growth was measured triplicate to detect the diameter of zone of inhibition and the mean were recorded. The lowest concentration of the tested isolated compounds that will inhibit the visible bacterial growth, minimal inhibitory concentration (MIC, μg mL^−1^) was determined as well [28].

### Antifungal assay

The antifungal activity of isolated compounds was assessed using well diffusion and broth microdilution techniques with positive control, Itraconazole. The tested pathogenic fungi were *Candida albicans* and *C. parapsilosis*. According to Gong and Guo [29], in SDA plate the sample solutions (100 µl), approximately 3 × 10^6^ colony-forming units (CFU) mL^−1^ was smeared. Wells were created in SDA plates and loaded with the 10 µg of the tested compounds. The plates were then incubated at 37 °C for 1 day. The diameters (in mm) of zone of inhibition were measured and the rates of growth inhibition were obtained according the following formula taking on consideration ± SD as means:

$$ \% {\text{Growth inhibition rate }} = \, \left( {d_{{\text{c}}} - d_{{\text{s}}} } \right) \, / \, \left( {d_{{\text{c}}} - d_{0} } \right) \, \times { 1}00 $$

where *d*_c_: Diameter of the untreated control fungus, *d*_s_: Diameter of the sample-treated fungus and *d*_0_: Diameter of the fungus cut.

## Conclusions

Polyketides possess a wide range of significant biological activities, such as anti-tumor, antimicrobial and anti-inflammatory. In our study, one new and four known metabolites were obtained from the large scale fermentation of the interrhizospheric fungus *Aspergillus* sp., and their antimicrobial activity was evaluated. The isolation of compounds **1**–**5** suggested that this *Aspergillus* strain is a powerful producer of polyketides with diverse structures. Compounds **1** showed significant antimicrobial activity against two pathogenic fungal strains *Candida albicans* and *C. parapsilosis* and a pathogenic strain of bacteria *Staphylococcus aureus* with MIC 2.3 μg mL^−1^. This study shows the importance of rhizospheric soil inhibited fungi as untapped source for novel secondary metabolites.

## Additional file

**Additional file 1.** NMR, Mass spectrum & chromatogram of extracts.

## Data Availability

All data and materials are fully available without restriction at the author’s institutions.
