# Supplementary material for: Secondary metabolites from the Aspergillus sp. in the rhizosphere soil of Phoenix dactylifera (Palm tree)
Source: BMC Chem. 2019 Aug 7;13(1):103. doi: 10.1186/s13065-019-0624-5 (PMC6686371; doi:10.1186/s13065-019-0624-5)
Supplement: Supplementary file 1 — Additional file 1. NMR, Mass spectrum & chromatogram of extracts. [file 13065_2019_624_MOESM1_ESM.docx]

Additional file 1

**Figure A1.** ^1^H NMR Spectra of Compound **1**.

**Figure A2.** ^13^C NMR Spectra of Compound **1**.

**Figure A3.** DEPT-135 NMR Spectra of Compound **1**.

**Figure A4.** HMBC Spectra of Compound **1**.

**Figure A5.** HSQC Spectra of Compound **1**.

**Figure A6.** Negative ESI Mass Spectra of Compound **1**.

**Figure A7.** Positive ESI Mass Spectra of Compound **1**.

**Figure A8.** The HPLC chromatogram for ASL (a) and ASS (b).

**Figure A1.** ^1^H NMR Spectra of Compound **1**.

**Figure A2.** ^13^C NMR Spectra of Compound **1**.

**Figure A3.** DEPT-135 NMR Spectra of Compound **1**.

**Figure A4.** HMBC Spectra of Compound **1**.

**Figure A5.** HSQC Spectra of Compound **1**.


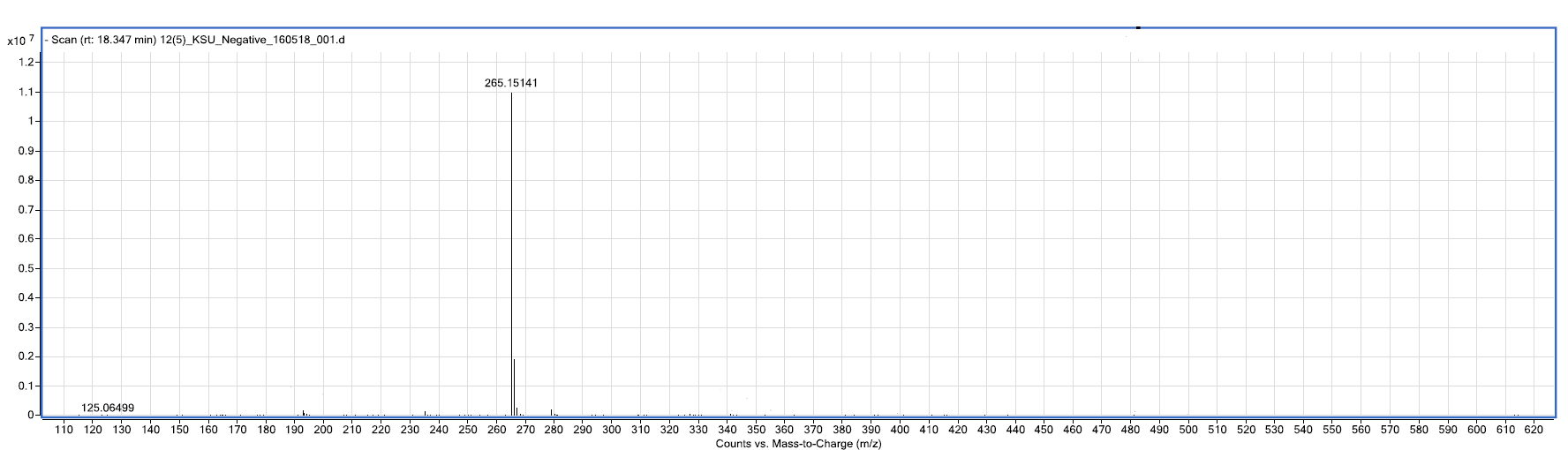


**Figure A6.** Negative ESI Mass Spectra of Compound **1**.


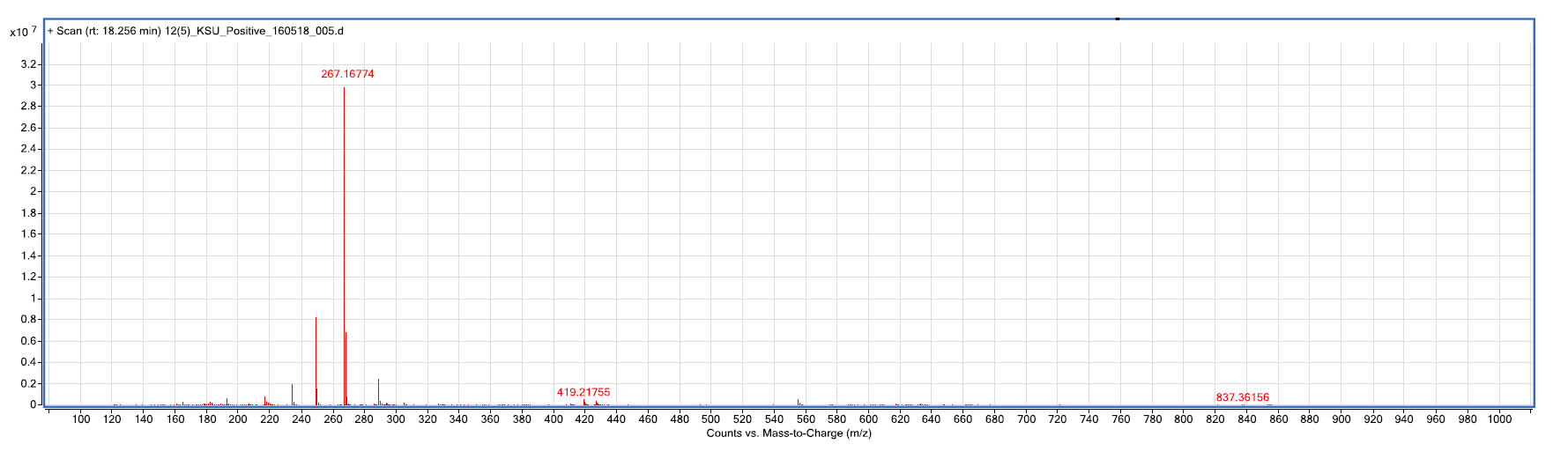


**Figure A7.** Positive ESI Mass Spectra of Compound **1**.

**(a)**

**(b)**

**Figure A8**: The HPLC chromatogram for ASL (a) and ASS (b).

***General Experimental Procedures***

Optical activity was measured on a JASCO P-2000 Series polarimeter (JASCO Corporation, 2967-5, Tokyo, Japan). The ^1^H, ^13^C NMR and 2D NMR spectra were recorded on a Bruker AMX-700 spectrometer with tetramethylsilane (TMS) as an internal standard. Chemical shifts are in ppm (δ), relative to tetramethylsilane as an internal standard and scalar coupling constants (J) reported in Hertz. ESI-MS analyses were measured on an Agilent Triple Quadrupole 6410 QQQ LC/MS mass spectrometer with ESI ion source (gas temperature is 350 °C, nebulizer pressure is 60 psi and gas flow rate is 12 L/min), operating in the negative and positive scanmodes of ionization through direct infusion method using CH3OH\H_2_O (1:1 v/v) at a flow rate of 0.2 mL/min. Column chromatography was carried out on silica gel and sephadex LH-20 (E. Merck, Darmstadt, Germany). Thin layer chromatography (TLC) was performed on precoated TLC plates (Aluminium sheets, silica gel & RP-18 F254, Merck, Germany); the detection was done at 254 nm and by spraying with ceric sulphate reagent. HPLC analysis was performed on a Prominence Shimadzu LC Solution, (Kyoto, Japan) and the system equipped with a CBM-20A communication bus module, two LC-10AD pumps, a CTO-10A(C) column oven, and an SPD 10A(V) diode array detector. A Shim-pack VP-ODS (150 mm×4.6 mm, 5.0 μm, Shimadzu) analytical column was used and kept at 40 °C. The mobile phase consisted of water containing 0.1% triflouroacetic acid (A) and CH_3_OH (B). The flow rate was set at 0.4 mL/min and the injection volume was 20 μL. The DAD detection was achieved in the range of 254 nm.
